# 1288. Rapidly evolving Lyme epidemiology in central Ohio creates challenges in prompt diagnosis and management of Lyme arthritis

**DOI:** 10.1093/ofid/ofad500.1127

**Published:** 2023-11-27

**Authors:** Guliz Erdem, Joshua R Watson, William J Barson

**Affiliations:** Nationwide Children's Hospital, Columbus, Ohio; Nationwide Children's Hospital, Columbus, Ohio; Ohio State University College of Medicine and Public Health and Nationwide Children's Hospital, Columbus, Ohio

## Abstract

**Background:**

Institutional practices regarding care for septic arthritis (SA) patients varies. To streamline the care of SA patients we established a clinical pathway with two care delivery expectations: % of patients on appropriate antibiotics and % of patients with time to diagnostic joint fluid aspiration < 24 hours after admission.

Key driver diagram

**Figure:**
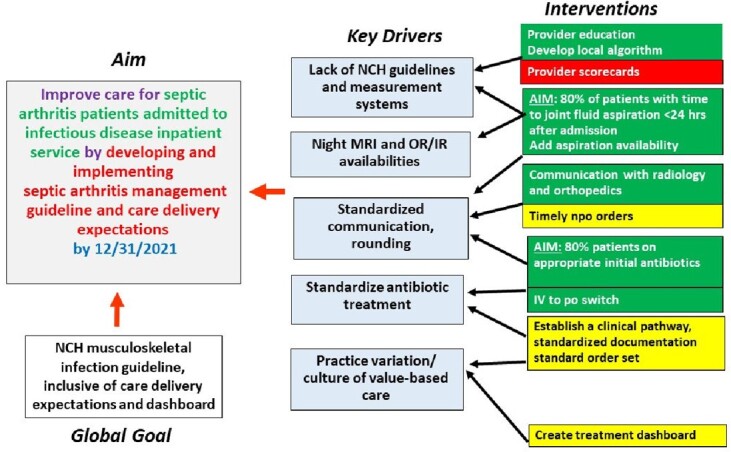

Lyme and non-Lyme bacterial arthritis patients since 2020

**Figure:**
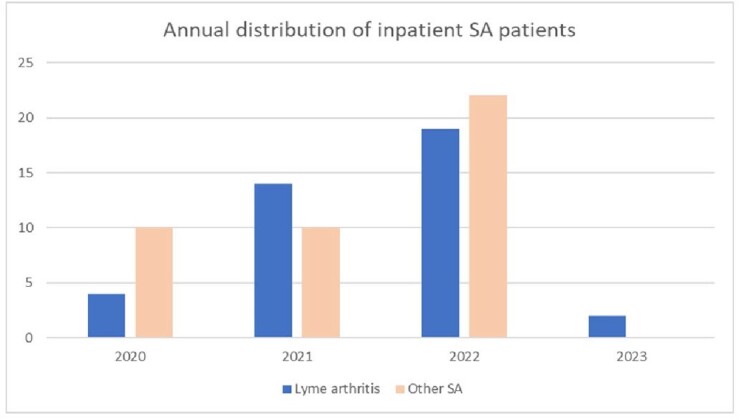

**Methods:**

Quality improvement initiative aimed to streamline the care and key drivers were established (Figure 1). Interventions included development management algorithm, consensus building among infectious diseases physicians. The pre-study baseline period was January 1, 2020 to May 31, 2021, and the study period was from June 1, 2021, to March 1, 2023. Control charts were used to assess the interventions.

**Results:**

Of the 97 patients admitted during the baseline and study period 42 patients were diagnosed with non-Lyme associated SA, 39 had Lyme arthritis, 16 had reactive arthritis. Significant increase in Lyme arthritis patients were observed since 2021 (Figure2) coinciding with reported geographic expansion of ticks in Ohio attributed to climate change. Length of hospitalization for Lyme arthritis (median: 2.35 days, IQR 1.29) was shorter than non-Lyme associated SA (median: 3 days, IQ 1.48). 18 (42.9%) of non-Lyme SA patients and 10 (25.6%) of Lyme arthritis patients underwent surgical washouts. 27 (69 %) of Lyme arthritis patients were initially treated with anti-staphylococcal antimicrobial agents. Lyme serologies took 1-3 days to result causing delays in diagnosis and management. 7 (18%) of patients were discharged home on no antibiotics or with antibiotics not appropriate for Lyme arthritis.

**Conclusion:**

Our streamlined approach led to timely and appropriate diagnostic and therapeutic approach in non-Lyme associated SA patients. Rapidly evolving Lyme epidemiology in central Ohio created challenges in prompt diagnosis and management of Lyme arthritis patients.

**Disclosures:**

**All Authors**: No reported disclosures

